# Guidelines for the pharmacological acute treatment of major depression: conflicts with current evidence as demonstrated with the German S3-guidelines

**DOI:** 10.1186/s12888-019-2230-4

**Published:** 2019-09-02

**Authors:** Martin Plöderl, Michael P. Hengartner

**Affiliations:** 1 Department for Crisis Intervention and Suicide Prevention, Christian Doppler Clinic, Paracelsus Medicial University, Ignaz Harrer Str. 79, 5020 Salzburg, Austria; 2 Zurich University of Applied Sciences, School of Applied Psychology, Zurich, Switzerland

**Keywords:** Depression, Pharmacological, Treatment, Antidepressants, Guidelines

## Abstract

Several international guidelines for the acute treatment of moderate to severe unipolar depression recommend a first-line treatment with antidepressants (AD). This is based on the assumption that AD obviously outperform placebo, at least in the case of severe depression. The efficacy of AD for severe depression can only be definitely clarified with individual patient data, but corresponding studies have only been available recently. In this paper, we point out discrepancies between the content of guidelines and the scientific evidence by taking a closer look at the German S3-guidelines for the treatment of depression. Based on recent studies and a systematic review of studies using individual patient data, it turns out that AD are marginally superior to placebo in both moderate and severe depression. The clinical significance of this small drug-placebo-difference is questionable, even in the most severe forms of depression. In addition, the modest efficacy is likely an overestimation of the true efficacy due to systematic method biases. There is no related discussion in the S3-guidelines, despite substantial empirical evidence confirming these biases. In light of recent data and with their underlying biases, the recommendations in the S3-guidelines are in contradiction with the current evidence. The risk-benefit ratio of AD for severe depression may be similar to the one estimated for mild depression and thus could be unfavorable. Downgrading of the related grade of recommendation would be a logical consequence.

## Background

Guidelines may be crucial for adequate treatment if they systematically and critically evaluate the evidence and infer treatment recommendations in a rational and transparent manner. This way, guidelines are an important interface between science and clinical practice. The obvious benefit of guidelines vanishes if the recommendations are misleading, for example because of biases in the synthesis of the evidence [1, 2], or simply because the evidence in the guidelines is outdated and conflicting with current evidence. Correcting the discrepancies between the content of the guidelines and current evidence is of utmost importance to avoid potentially harming patients. This seems to be the case for the acute pharmacological treatment of unipolar depression (synonymous to major depression), as we demonstrate in this article. We will mainly focus on the German S3-guidelines from 2015 (with updates until March 2017) [3]. However, algorithms in other guidelines are largely comparable, for example in the guidelines of organizations such as RANZCP (Australia and New Zealand) or NICE (UK) [4, 5], thus our findings are relevant beyond Germany.

## Methods

We reviewed the sections of the S3-guidelines about the acute pharmacological treatment of unipolar depression (sections 3.4.1. to 3.4.4) with two objectives. First, we investigated if the data about the efficacy of antidepressants (AD) is still in line with current meta-analytic evidence, and also if the clinical importance of the findings is discussed. Since main arguments of the treatment recommendations rely heavily on the efficacy of AD for different levels of depression severity, we included a simple systematic review of related efficacy studies based on individual patient data. We therefore systematically searched PubMed on November 21, 2018, using the following terms: (“individual participant” OR “individual patient” OR “participant level” OR “patient level” OR “individual level”) AND (“meta” OR “meta-analysis”) AND (depression OR SSRI OR SNRI OR antidepressants OR “mood disorder” OR “affective disorder”). This resulted in 185 hits. After screening the abstracts, 149 studies could be excluded because they obviously did not include relevant information. The remaining 36 studies were screened in detail and 10 studies included primary information of interest [6–15]. We also checked the references of these studies and could find one more relevant study [16]. The 11 relevant studies are summarized in Table 2. The second objective was to review if empirically supported method-biases were adequately addressed as limitations in the judgment of the evidence [17].

## Results and discussion

### Efficacy of antidepressants

#### Comparing the evidence in the guideline with current evidence

In the S3-guidelines, the efficacy of antidepressants (AD) in the acute treatment of major depression is summarized as follows [3]:

*To prove a clinically relevant efficacy of acute antidepressant treatment in placebo-controlled trials, a minimum improvement of 50% on established scales (e.g., the Hamilton Rating Scale) is suggested […] In these kinds of clinical trials with a maximum duration of up to twelve weeks, the response rates mostly range between 50 and 60%, the placebo response rates about 25–35% (p. 67)*.^1^

Thus, the difference in response rates between AD and placebo is reported to be around 25%. This conclusion is based on two outdated studies; a meta-analysis and a review [18, 19]. The 25%-difference contradicts the results from current meta-analyses which reported a difference of about 10% [20, 21], with response rates of approximately 50 and 40% for AD and placebo, respectively (Table 1). A common counter-argument is that response rates for placebo have increased over the years, leading to decreasing AD-placebo differences. This argument is often based on an outdated meta-analysis of Walsh et al. from 2002 [19]. However, a recent meta-analysis found that the placebo-response rates did not increase from 1991 onwards [22]. Therefore, the 25–35% placebo response rate and the approximately 25% difference in response rates between AD and placebo reported in the S3-guidelines substantially deviate from the current evidence.

**Table 1:** Meta-analyses about the efficacy of AD compared to placebo

	Response Rates (at least 50% reduction in depression)		
	AD (%)	Placebo (%)	Difference
S3-guidelines summary statement on efficacy these were based on:	50–60	25–35	ca. 25
1. Walsh et al. (2002) [19]	50	30	20
2. Oeljeschläger et al. (2004) [18]^a^	67	47	20
Current Meta-Analyses			
Cipriani et al. (2018) [20]^b^	ca. 50	ca. 40	ca. 10
Jakobsen et al. (2017) [21]^c^	49	39	10
Meta-Analyses available before the last update of the S3-guidelines			
Furukawa et al. (2016) [23]		35–40	
Weitz et al. (2015) [24]	42 (Duloxetine) 45 (SSRIs)	24	18–21
Nelson et al. (2013) [7]	49	40	9
Gibbons et al. (2012) [6]			
mild depression	55	37	18
severe depression	58	41	17
Undurraga & Baldessarini (2012) [25]	54	37	17
Melander et al. (2008) (SSRI + SNRI) [26]	48	32	16
Arroll et al. (2005) [27]	SSRI: 56	41	15
	TCI: 60	47	13
Storosum et al. (2004) (only TCA) [28]	39	28	11

Notes

^a^ This review claims a “far-reaching agreement” that two-third respond when treated with AD, whereas there are 20% less responders under placebo, referencing a review of Bauer et al. (2002). The Bauer et al. review, in return, reported a response rate of 50–75% for the old generation AD for medium to severe depression and of 25–33% for placebo (based on a review of the American Psychiatric Association from the year 2000), as well as a response rate of 50% for SSRIs and of 32% for placebo (based on a report from the Agency for Health Care Policy and Research from the year 1999). Thus, the conclusions not only deviate from the cited sources, but these sources are also outdated, since they were published at least 15 years before the publishing of the S3-guidelines

^b^ Cipriani et al. did not report response rates, but they were estimated elsewhere [29], using an average effect of OR = 1.66 and a response rate of 30–40% for placebo. We also tried to estimate the difference between the AD and placebo response rates, using the results from Jakobsen et al. (2017) [21] who reported 39% responders under placebo. With the average effect of OR = 1.66, we came up with nearly identical results (51% responders under AD and 39% under placebo). Formula: R_AD_ = OR*R_p_/(1-R_p_ + OR*R_p_). R_AD_: response rate AD, R_p_: response rate placebo

^c^ based on the results for nonresponse

We also noted a discrepancy between the summary statement regarding the efficacy of AD (50–60% responders on AD as compared to 25–35% on placebo) and the two studies that were cited in support of this statement [18, 19]. One study [18] claimed that “there is a far-reaching agreement” that two-third of patients respond to AD, but this is not supported by the referenced evidence (Table 1). Furthermore, both cited studies reported differences in response rates between AD and placebo of only 20% and not 25%. In addition, it is surprising that the S3-guidelines did not include meta-analyses that were already available before the guidelines were updated and published [6, 7, 23–28] (see Table 1). These newer meta-analyses found substantially lower differences in response rates between AD and placebo than the reported 25%, and also much higher placebo response rates. Thus, even without the latest meta-analyses published after 2017, the overall assessment of efficacy should have been different.

The impression of an exaggerated presentation of the efficacy of AD also occurs in the discussion of the efficacy of different types of AD. For SSRIs, the following is claimed:

*The group of selective serotonin-reuptake-inhibitors (SSRI) […] increases the central serotonergic neurotransmission by selectively inhibiting the reuptake of serotonin from the synaptic cleft. This explains the antidepressant effects as well as the side effects. The efficacy of selective serotonin reuptake inhibitors (SSRIs) in the treatment of acute depressive episodes has been demonstrated in many clinical studies* versus *placebo and in corresponding meta-analyses. (p. 69).*

Some of the SSRI-trials cited in the S3-guidelines reported rather small effect-sizes and this should have raised doubts on the summary efficacy statement mentioned above. More importantly, the largest and most recent meta-analysis cited in the S3-guidelines [27] reported a high response rate for placebo (41–47%), which grossly deviates from the summary statement (25–35%).

One reason why recent meta-analyses reported smaller differences between AD and placebo lies in the fact that they were based on both published and unpublished studies, whereas earlier meta-analyses exclusively relied on studies published in scientific journals [20, 21, 30]. A related well known publication bias is that positive studies were almost always published in scientific journals (sometimes multiple times), but negative trials were rarely published [31, 32]. According to a comprehensive analysis of the trial-results available to the FDA, only 51% of studies were positive and 97% of these studies were published as positive studies in journals. In contrast, only 3% of negative studies were published as being negative in a journal. Furthermore, 21% of negative studies were published as being positive, for example by only reporting on a secondary outcome that was then falsely reported to be the primary outcome, or by only reporting the results of a subgroup. All other negative studies remained unpublished [32]. Thus, despite that only about half of the AD-trials were positive, nearly all related published studies report positive findings [33]. This important bias is briefly mentioned in the S3-guidelines, but the implications are not considered any further in the evaluation of the evidence from published AD trials.

One common explanation for the modest efficacy of AD in more recent studies is that there is a trend to only include less severely depressed patients or those without frequent prior depressive episodes [5] (p. 308). However, this does not seem to be the case, instead, it was the rate of drop-outs due to inefficacy in placebo-groups that has changed [34]. The average drop-out rate in the year 1985 was 58% and of those who discontinued the studies early, 93% stated lack of efficacy as a reason. In the year 2009, only 20% of patients in the placebo-group dropped out, and only 15% attributed this to lack of efficacy [34]. The massive reduction of placebo-dropouts due to lack of efficacy is crucial, because this can fully explain the reduced efficacy of AD in more recent studies. Moreover, this effect appears to be robust and consistent, as it is independent of the length of the study or sample-size. Thus, instead of the typical explanation that the placebo-response is miraculously greater in more recent studies, a more accurate interpretation is that patients on placebo do not immediately drop-out if they do not recognize some effect of the drug [34] (this also raises the question of successful blinding of patients and doctors in older trials). Since patients could be kept longer in more recent studies, it seems that substantially more patients in the placebo-group achieve spontaneous remission until the end of the trial, leading to a reduction of the difference between AD and placebo, even when they may not perceive a drug effect.

#### Discussion of clinical significance

There is a controversy about the appropriateness of using response rates, because this can lead to an overestimation of the efficacy of a treatment [35] (also see footnote 2). This problem is briefly mentioned in the S3-guidelines:

*Furthermore, the efficacy in comparison to placebo is mostly based on the higher response rate, whereas the difference in remission-rates or the reduction of summary-scores of depression rating-scales is often not significant (p. 67).*

However, it is not discussed what “not significant” actually implies. In the meantime, it has been replicated many times that even though the AD-placebo difference is *statistically* significant, this effect may not be *clinically* significant [17, 21, 36]. This was already discussed in publications available at the time well before the S3-guidelines were published [35, 37, 38]. For example, Kirsch and colleagues demonstrated that most variance (> 75%) in the outcome in the SSRI groups can be attributed to placebo-responses, and the rest may result from enhanced placebo responses due to perceived side-effects of AD [37]. According to the most recent meta-analysis of Cipriani and colleagues [20], the overlap between AD and placebo is even larger (88%) [17, 39].

Admittedly, there is no universal definition of “clinical significance” (see Footnote 2). However, AD do not meet any criterion for clinical significance, not even the most liberal [17, 39]. This is not surprising, because the average difference of AD compared to placebo is only about 2 points on the HAMD-17 depression rating scale that has a range from 0 to 52 points (most items are scored between 0 and 4). This is intuitively a very modest and unimportant effect, which is also confirmed when the 2 point difference is compared to clinical judgments made by mental health professionals. If the HAMD is compared to the clinical evaluation using the Clinical Global Impression Improvement Scale (CGI-I), then 0–3 points improvement on the HAMD correspond to “no improvement” on the CGI-I. It needs at least 7 points improvement on the HAMD scale to achieve a corresponding “minimal improvement” on the CGI-I. None of the AD come anywhere near this criterion [17].

Furthermore, the S3-guidelines seem to have a contradictory use of clinical significance, because it is questioned in one section and then taken for granted in other sections. When the efficacy of AD for mild depression is discussed (p. 68), the criterion of 3 HAMD-points for clinical significance is questioned with the argument that this criterion was removed from the current NICE guidelines. This is wrong, because the NICE guidelines from 2010 did include this criterion in an appendix [5].^2^ Doubts on the criterion for clinical significance also appear when discussing a study which reported less than 3 HAMD-points difference between AD and placebo for both mild and more severe depression [6]. Interestingly, this important study is then ignored in the following section (also p. 68) about the treatment of moderate to severe depression. Instead, it is stated that for severe depression, AD are clinically superior to placebo, based on the 3-point criterion for clinical significance.

#### Efficacy of AD in relation to depression severity – guidelines versus current evidence from a systematic review

The S3-guidelines report that, for mild depression, AD are not superior to placebo, resulting in an unfavorable negative risk-benefit ratio because of the side-effects of AD. The NICE guidelines include very similar arguments: “Do not use antidepressants routinely to treat persistent subthreshold depressive symptoms or mild depression because the risk-benefit ratio is poor (p. 327)” [5]. Likewise, the RANZCP guidelines recommend that “patients with mild-moderate depression should be offered one of the evidence based psychotherapies as first line treatment” (p. 1108) [4] (the negative risk-benefit ratio is not explicitly stated but the logical argument behind this conclusion is given).

For moderate to severe depression, the S3-guidelines report that AD have a clinically significant effect:

*For medium to severe depression, however, the difference in efficacy between antidepressants and placebo is more pronounced, since in the most severe forms up to 30% of treated patients benefit from antidepressants above the placebo rate. Thus, HDRS scores of > 24 are associated with the most consistent difference between the response to drug and placebo, whereby these differences in the direction of the active antidepressant are also clinically significant (p. 68).*

This statement is based on a single citation, referring to a study by Khan et al. (2005), but this study is not related to depression at all and is most likely a citation error. We guess that the authors of the S3-guidelines wanted to refer either to another publication of Khan [40], or to the meta-analysis of Fournier et al. [9] that is frequently cited in this context.

To clarify if AD are more efficacious for severely depressed patients, individual-level data from patients are needed, because using group means leads to substantial biases (referred to as ecological fallacy) [41]. It is surprising that this argument is completely lacking in the S3-guidelines, even more so, as two such studies with individual patient data were cited in the S3-guidelines, and these studies addressed the problems resulting from group-level data [6, 9]. In addition, one of these studies did not find AD to be clinically effective for severe depression [6], but this study was not discussed appropriately, as we already noted above.

Our simple systematic review of studies with individual patient-level data could locate 11 relevant studies that are summarized in Table 2. It can be concluded that most patient-level meta-analyses, especially the more recent and larger ones, reported that AD are not clinically significantly superior to placebo, even for severe depression (< 3 HAMD-points difference between AD and placebo). One exception is a study in older patients, where one subgroup (severely and chronically depressed patients) responded much better to AD than to placebo [7]. However, this could be a false positive finding because of multiple testing of many different subgroups. Also, according to the meta-analysis of Fournier et al. [9], AD were substantially more efficacious than placebo in patients with a baseline score of ≥23 on the HAMD, but this was refuted in recent and larger meta-analyses. One very recent study reported that placebo is slightly more effective than AD for the most severely depressed patients [15]. Finally, it was also found that AD were not more efficacious for the melancholic subtype of depression – which is associated with higher depression-scores and seen as the most severe form of depression by many experts [12].

**Table 2:** Meta-analyses based on individual patient data

Study	Sample characteristics	Results	Are AD clinically significant for severe depression?
Thase et al. (2007) [8]	6 placebo-controlled studies, 1833 patients	Remission rates, statistical significance and size of the interaction term (depression severity × treatment group) not reported HAMD 15–18: duloxetine 46.5%, SSRI‘s: 51.7%, placebo: 42.7% HAMD ≥19: duloxetine 35.9%, SSRI‘s: 28.6%, placebo: 17.7%	Yes, but not definitely
Fournier et al. (2010) [9]	Systematic review, 6 studies (paroxetine, imipramine), 434 patients in AD groups, 284 in placebo groups	Mild to moderate depression (HAMD ≤18): d = 0.11 (0.9 HPD)^a^ Severe depression (HAMD 19–22): d = 0.17 (1.4 HPD) Very severe depression (HAMD ≥23): d = 0.7 (3.8 HPD)	Yes
Khan et al. (2011) [10]	15 trials of one center, 262 patients treated with AD, 140 with placebo	HAMD score was a significant predictor of a reduction of depression scores for patients treated with AD, but not so for patients in the placebo groups. However, the statistical significance and the size of the interaction term (depression severity × treatment group) is not reported	?
Gibbons et al. (2012) [6]	Fluoxetine studies (Eli Lilly & Co), one study on adolescents, venlafaxine studes (Wyeth), total of 31 studies and 9185 patients	HAMD ≤20: 2.2 HPD HAMD > 20: 2.8 HPD Similar results for different AD and age-groups	No
Nelson et al. (2013) [7]	Second generation AD, 10 studies with 2283 older patients (≥60 years)	Significant effect only for the AD group. No statistically significant interaction between depression severity and treatment in the multivariate analysis. Differences of response rates for HAMD > 23 ≈ 18%, for HAMD 21–23 ≈ 8%, for HAMD 19–20 ≈ 12%, and for HAMD < 19 ≈ 0%. No mean-values are reported, except for the chronically depressed subgroup (d ≈ 0.7 for HAMD > 23 (5.6 HPD), d ≈ 0.4 (3.2 HPD) for HAMD 21–23, d < 0.1 (0.8 HPD) for HAMD < 21.	? or only in one subgroup
Harada et al. (2015) [11]	4 studies with duloxetine and different SSRIs, total of 1694 patients	HAMD ≥15:1.4–1.5 HPD HAMD ≥19: 2.1–2.2 HPD	No
Rabinowitz et al. (2016) [16]	34 studies with second generation AD or quetiapine (4 studies), total of 10,737 patients	HAMD < 22: 2.04 HPD HAMD 22–25: 1.82 HPD HAMD > 25: 2.41 HPD	No
Cuijpers et al. (2017) [12]	4 studies, total of 333 patients, SSRI vs. placebo vs. psychotherapy	Comparison of melancholic depression (with an increased HAMD score of about 1.5 points) with other types of depression. No significant interaction effects (0.53 HPD melancholic type vs. 0.33 HPD for other types of depression)	No
Debray et al. (2018) [13]	18 studies of older generation AD vs. Placebo, 2456 patients	HAMD = 21.8: 2.2 HPD HAMD = 25: 3.1 HPD	?
Furukawa et al. (2018) [14]	Systematic review of pre-registered Japanese trials, 6 studies and 2464 patients	No significant interaction of depression severity and treatment group. Ca. 1.6 HPD across the whole spectrum of depression severity	No
Nakabayashi et al. (2018) [15]	5 studies used for approval of AD in Japan, 1898 patients	No significant interaction of depression severity and treatment group. HAMD 8–13: − 0.36 HPD; HAMD 14–18: − 1.50 HPD. HAMD 19–22: 3.60 HPD; HAMD ≥23: − 1.26 HPD	No for most severely depressed, yes for HAMD 19–22

Notes

A negative point-difference means that placebo is more effective than AD

After submitting a revised version of our manuscript, a large patient-level meta-analysis was published (Hieronymus et al., 2019, https://doi.org/10.1016/S2215-0366(19)30216-0). In this study, despite excluding patients post-hoc, the HPD was consistently less than 3 HAMD-17 points across the whole severity spectrum. This was not explicitly mentioned in the paper, but can be inferred from the results.

The transformation of Cohen’s d into HAMD-point-differences was based with an assumed standard deviation of SD = 8 [17, 42]

^a^HPD: Difference of HAMD points

#### Discussion of method biases

The S3-guidelines did not include a discussion of important biases, except for the publication bias:

*In the perception of the (specialist) public, the efficacy of antidepressants is rather overestimated, since studies in which the antidepressant performed better than placebo are published much more frequently in scientific journals than those in which the antidepressant was not superior to placebo (p. 67).*

So the publication bias is briefly mentioned, but it was not considered elsewhere. This is problematic in sections where treatments were compared with each other, based on single or very few published studies. Due to the publication and sponsorship bias, where negative results are rarely published, these comparisons are likely biased [43]. Moreover, throughout the guidelines, the efficacy of different treatment approaches is often based on statistical significance alone. It is known that statistical significance is not informative about the size of a difference or about clinical significance [39].

There are many more biases that may lead to an overestimation of the efficacy of AD, but they were not discussed in the S3-guidelines. Such biases include unblinding due to specific side-effects of AD, exclusion of patients who improve in the placebo lead-in phase, withdrawal effects in the placebo group due to abrupt discontinuation of pre-trial AD prescriptions, inadequate handling of missing data with last observations-carried forward, and other biases [44–46]. Some of these biases, for example the breaking of the double-blinding due to correct guessing of placebo or drug, have been replicated in various empirical studies and are known for a long time [47, 48]. There is also sound evidence that unblinded physicians judge the drug as being more effective than blinded physicians [49, 50]. Just recently, it was found that trials with a placebo lead-in phase produce significantly larger efficacy estimates than the minority of trials without such a lead-in phase (d = 0.31 vs. d = 0.22) [51]. This was long expected by various experts, because patients who improve during the placebo lead-in phase are excluded from the trial, biasing the results in favor of AD. Thus, it can be concluded with a high degree of certainty, that the efficacy of AD is overestimated in typical clinical trials. In contrast, we are not aware of empirical studies confirming postulated biases leading to an underestimation of the efficacy of AD [52, 53]. On the contrary, some of these biases were refuted in the meantime. For example, it is often claimed that AD work much better in real-world patients. However, AD are no more effective in patients treated in the real-world routine practice compared to those selected for clinical trials, as clearly demonstrated in the STAR*D study [54, 55] or in a meta-analysis of real-world primary care patients [56]. Some other assumed biases do not seem very plausible, for example the argument that patients lie about their depression to be included in studies in order to obtain treatment for free or to receive some money. Even if this is so, there is no plausible explanation as to why this should lead to biased drug-placebo differences, since these malingerers would be randomly assigned to treatment arms. In any case, there is no empirical evidence that would support such an assumption, and as such it is no more than an untested hypothesis. Another popular argument is that some trials allow additional treatment with benzodiazepines and other tranquilizers, but this would affect both the AD and the placebo groups similarly, so this is no systematic bias and both direction and size of the bias are still unknown.

## Conclusions

The S3-guidelines and other international guidelines do not recommend AD as first-line treatment for mild depression, because:

*Due to the unfavorable risk-benefit ratio, antidepressants are not generally useful in the initial treatment of mild depressive episodes, since antidepressant medication is hardly superior to a placebo condition (p. 74, citations removed).*

As we have shown in this paper and discussed elsewhere [17, 39], AD are indeed hardly superior to placebo in mild depression, but the same holds for moderate and severe depression (i.e., less than three points on the HAMD scale or approximately 10% difference in response rates). This already modest efficacy is most likely an overestimation of the true effect size due to various systematic method biases inherent in clinical trials. Therefore, the degree of recommendation for the pharmacological acute treatment of moderate and severe depression with AD should be downgraded on the basis of the guidelines’ own logic. We are not alone with such conclusions. Munkholm et al. [51] recently re-analyzed the trial data for moderate to severe depression collected by Cipriani et al. [20], and based on the poor efficacy estimates and the many systematic biases in these trials, they concluded that “the evidence does not support definitive conclusions regarding the efficacy of antidepressants for depression in adults, including whether they are more efficacious than placebo” (p. 8). Consequently, this impacts the risk-benefit ratio of AD in the acute treatment of major depression, as well as comparisons of AD with alternative treatments. Therefore, treatment recommendations should be critically discussed in light of the current evidence. This clearly goes beyond the scope of this paper, but good examples are available [57]. We hope that our review can inform clinicians until the guideline will be updated accordingly.

## Data Availability

The list of studies of the systematic review and additional information can be found here: https://osf.io/4kh2a/

## Abbreviations

AD: Antidepressant(s)
BDI: Beck Depression Inventory
CGI-I: Clinical Global Impression Scale
d: Cohen’s d
FDA: Food and Drug Administration
HAMD or HDRS: Hamilton Depression Rating Scale
HAMD-17: Hamilton Depression Rating Scale, version with 17 items
HPD: Hamilton Rating Scale points difference between AD and Placebo
NICE: National Institute for Health and Care Excellence
OR: Odds-ratio
RANZCP: Royal Australian and New Zealand College of Psychiatrists
RCT: Randomized controlled trial
RR: Risk-ratio
SNRI: Serotonin-noradrenaline reuptake inhibitor
SSRI: Selective serotonin reuptake inhibitor
TCA: Tricyclic Antidepressants

## Footnotes

1 All quotations from the S3-guidelines were translated into English by the authors.

2 Unfortunately, the NICE guidelines did not justify the different definitions of clinical significance. Three different criteria for clinical significance were defined: First, a ≥ 3 points difference between AD and placebo on the HAMD scale (or the BDI scale). Second, an effect-size of d ≥ 0.5 (equivalent to approximately 3.8 points difference on the HAMD scale [17]). Third, a risk-ratio (RR) of RR ≤ 0.8 for response rates. Of note, these criteria are an absolute minimum, corresponding to a “no improvement” clinical judgment, but this is not mentioned. Furthermore, the three criteria are not equivalent, leading to contradictory conclusions. For example, the average effect-size in a recent meta-analysis [20] was d = 0.3 (clearly below the required d = 0.5), corresponding to a 2.4 HAMD points difference (below the required 3 points), but to a risk ratio of RR = 0.8 (only just fulfilling the criterion).
